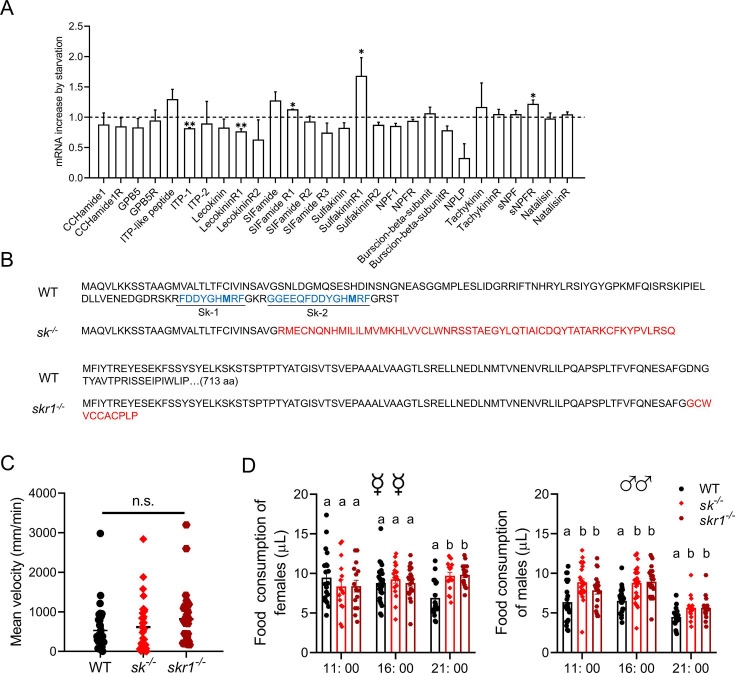# Correction: The neuropeptide sulfakinin is a peripheral regulator of insect behavioral switch between mating and foraging

**DOI:** 10.7554/eLife.112748

**Published:** 2026-07-28

**Authors:** Hong-Fei Li, Bao Dong, Yuan-Yuan Peng, Hao-Yue Luo, Xiao-Lan Ou, Zheng-Lin Ren, Yoonseong Park, Jin-Jun Wang, Hongbo Jiang

**Keywords:** Other

Li H-F, Dong B, Peng Y-Y, Luo H-Y, Ou X-L, Ren Z-L, Park Y, Wang J-J, Jiang H-B. 2025. The neuropeptide sulfakinin is a peripheral regulator of insect behavioral switch between mating and foraging. *eLife* **13**:RP100870. doi: 10.7554/eLife.100870.

Published 2 May 2025

A reader kindly alerted us to concerns regarding the source data for Figure 2D (Figure 2—source data 1) in the original publication. Specifically, the reader noted that (i) the feeding data for the sk^⁻/⁻^ group at 21:00 and the skr1^⁻/⁻^ group at 21:00 were identical (values were duplicated) but presented in a different sequence, (ii) some calculated feeding values appeared more frequently than expected, and (iii) all the data for Figure 2D end with "9".

We compared the published source data with our original records and confirm that our local raw data for skr1^⁻/⁻^ at 21:00 are unaffected by the data duplication error. The errors in the published Source data resulted from selecting and converting the wrong raw OD values to calculate the food consumption for the skr1^⁻/⁻^ group at 21:00. Raw OD values for skr1^⁻/⁻^ at 21:00 correspond to the OD values for sk^⁻/⁻^ at 21:00, which was an accidental copy-and-paste error due to converting the complicated data layout generated by the microplate reader. Whilst reviewing our original records, we also found that the raw data for female fruit flies (left panel of Figure 2D) was missing from the published source data.

As for the fact that all values in the source data file for Figure 2D end with the digit “9”. This is not an anomaly but a mathematical consequence of the different decimal places used in our calculation. The feeding amount is calculated from the OD value by a linear regression function generated from a standard curve: Y=101.4 X+0.004828. X = (OD of fly supernatant) – (OD of buffer). We used the average buffer OD value (0.05067, previously kept to 5 decimal places). However, the measured OD values for fly samples are read to 3 decimal places (as given by the plate reader). To keep accordance with the measured samples and avoid any misunderstanding, we kept all the computed values to 3 decimal places in the corrected Figure 2—source data 1. This change does not affect the data size or the corresponding bar chart.

We also corrected Figure 2—source data 1 to include the previously omitted raw feeding data for female flies (left panel of Figure 2D), corrected the raw data for skr1^⁻/⁻^ male flies at 21:00, and corrected the bar chart in Figure 2D (right) for skr1^⁻/⁻^ male flies at 21:00.

The manuscript’s results and conclusions remain unaffected by these errors. This is because its findings and text were based on the local original data. No corrections to the text were needed.

The corrected Figure 2 (updated for panel D, bar chart for skr1⁻/⁻ male flies at 21:00) is shown here:

**Figure 1:**
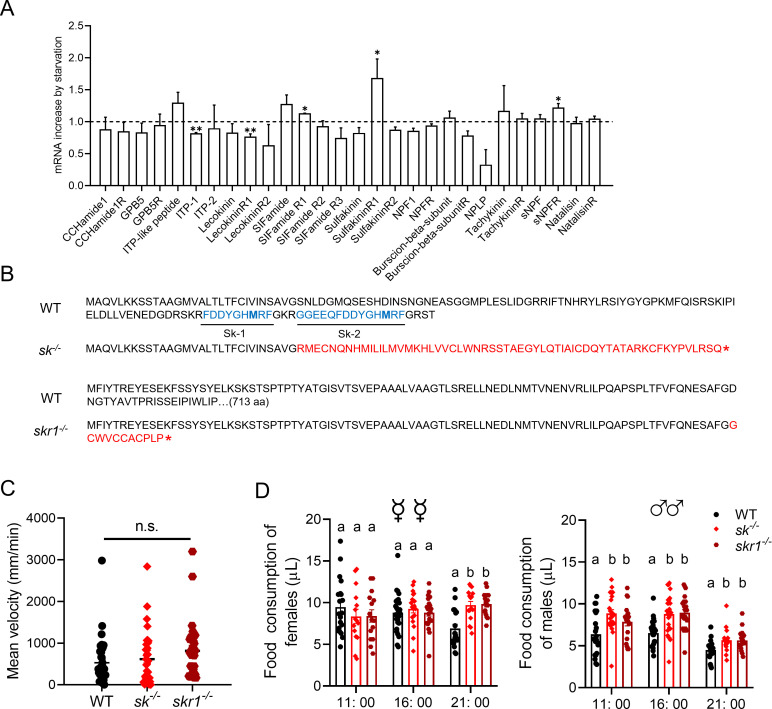


The originally published Figure 2 is shown for reference:

**Figure 2:**