# Supplementary material for: The neuropeptide sulfakinin is a peripheral regulator of insect behavioral switch between mating and foraging
Source: eLife. 2025 May 2;13:RP100870. doi: 10.7554/eLife.100870 (PMC12048153; doi:10.7554/eLife.100870)
Supplement: Supplementary file 3. [file elife-100870-supp3.docx]

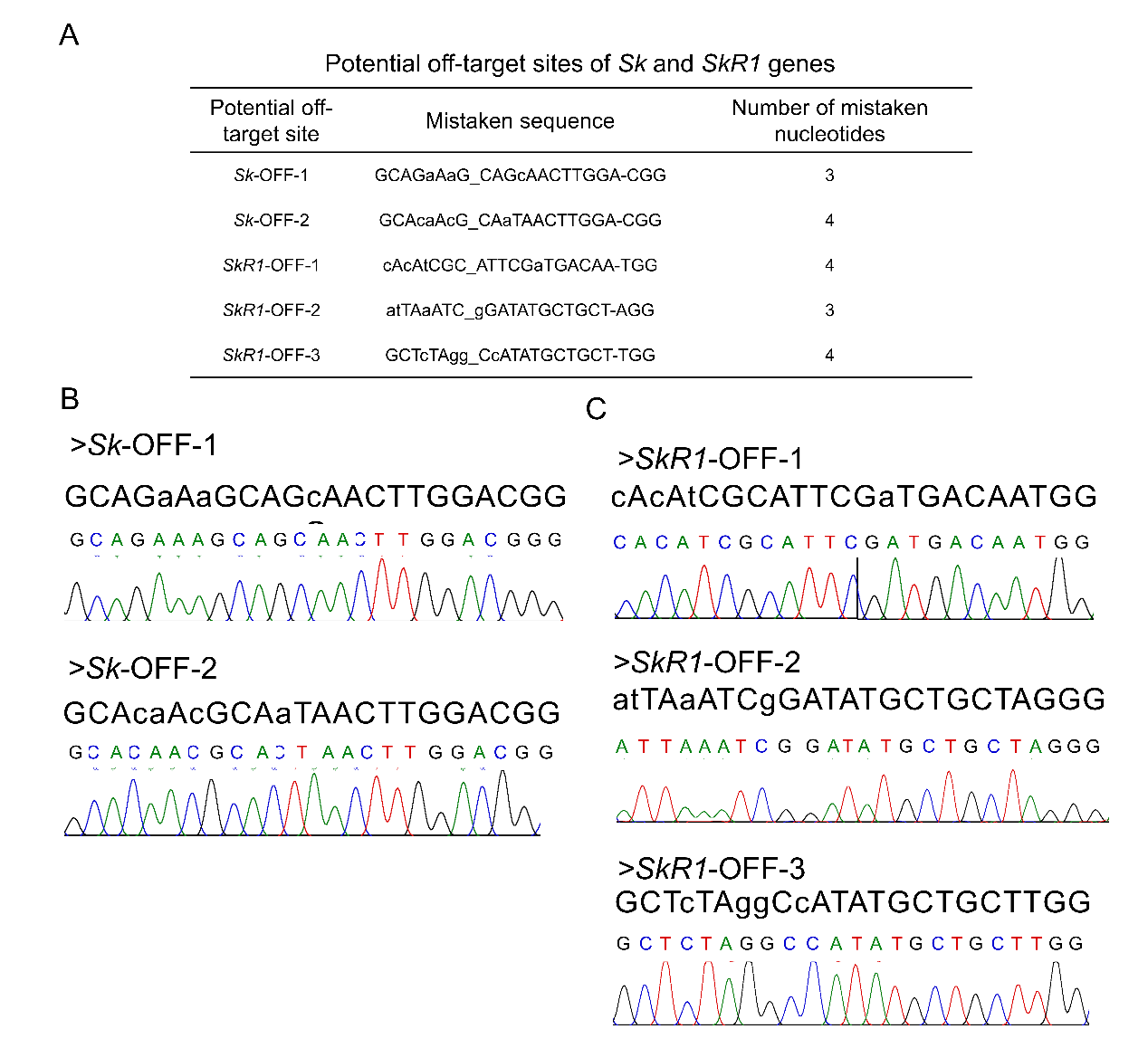


**Supplementary File 3.** Analysis of off-target effects of mutants. (*A*) Prediction of potential off-target sites of the *Sk* and *SkR1* genes. (*B*) PCR amplification and sequencing of potential off-target sites of the *Sk* gene. (*C*) PCR amplification and sequencing of potential off-target sites of the *SkR1* gene.
